# Physicochemical and Biological Characterisation of Diclofenac Oligomeric Poly(3-hydroxyoctanoate) Hybrids as β-TCP Ceramics Modifiers for Bone Tissue Regeneration

**DOI:** 10.3390/ijms21249452

**Published:** 2020-12-11

**Authors:** Katarzyna Haraźna, Ewelina Cichoń, Szymon Skibiński, Tomasz Witko, Daria Solarz, Iwona Kwiecień, Elena Marcello, Małgorzata Zimowska, Robert Socha, Ewa Szefer, Aneta Zima, Ipsita Roy, Konstantinos N. Raftopoulos, Krzysztof Pielichowski, Małgorzata Witko, Maciej Guzik

**Affiliations:** 1 Jerzy Haber Institute of Catalysis and Surface Chemistry Polish Academy of Sciences, Niezapominajek 8, 30-239 Kraków, Poland; tomasz.witko@ikifp.edu.pl (T.W.); Malgorzata.zimowska@ikifp.edu.pl (M.Z.); Robert.socha@ikifp.edu.pl (R.S.); malgorzata.witko@ikifp.edu.pl (M.W.); 2 Faculty of Materials Science and Ceramics, AGH University of Science and Technology, 30 Mickiewicza Ave., 30-059 Kraków, Poland; ecichon@agh.edu.pl (E.C.); skibinski@agh.edu.pl (S.S.); azima@agh.edu.pl (A.Z.); 3 Faculty of Physics, Astronomy and Applied Computer Science, Jagiellonian University, Lojasiewicza 11, 30-348 Kraków, Poland; daria.solarz@doctoral.uj.edu.pl; 4 Department of Physical Chemistry and Technology of Polymers, Silesian University of Technology, M. Strzody 9, 44-100 Gliwice, Poland; Iwona.Kwiecien@polsl.pl; 5 School of Life Sciences, College of Liberal Arts and Sciences, University of Westminster, New Cavendish Street, London W1W 6UW, UK; w1614733@my.westminster.ac.uk; 6 Department of Chemistry and Technology of Polymers, Cracow University of Technology, Warszawska 24, 31-155 Kraków, Poland; ewa.szefer@doktorant.pk.edu.pl (E.S.); konstantinos.raftopoulos@pk.edu.pl (K.N.R.); kpielich@pk.edu.pl (K.P.); 7 Department of Materials Science and Engineering, University of Sheffield, Broad Lane, Sheffield S3 7HQ, UK; i.roy@sheffield.ac.uk

**Keywords:** tricalcium phosphate composites, polyhydroxyalkanoates, polyhydroxyoctanoate oligomers, diclofenac, bone regeneration

## Abstract

Nowadays, regenerative medicine faces a major challenge in providing new, functional materials that will meet the characteristics desired to replenish and grow new tissue. Therefore, this study presents new ceramic-polymer composites in which the matrix consists of tricalcium phosphates covered with blends containing a chemically bounded diclofenac with the biocompatible polymer—poly(3-hydroxyoctanoate), P(3HO). Modification of P(3HO) oligomers was confirmed by NMR, IR and XPS. Moreover, obtained oligomers and their blends were subjected to an in-depth characterisation using GPC, TGA, DSC and AFM. Furthermore, we demonstrate that the hydrophobicity and surface free energy values of blends decreased with the amount of diclofenac modified oligomers. Subsequently, the designed composites were used as a substrate for growth of the pre-osteoblast cell line (MC3T3-E1). An in vitro biocompatibility study showed that the composite with the lowest concentration of the proposed drug is within the range assumed to be non-toxic (viability above 70%). Cell proliferation was visualised using the SEM method, whereas the observation of cell penetration into the scaffold was carried out by confocal microscopy. Thus, it can be an ideal new functional bone tissue substitute, allowing not only the regeneration and restoration of the defect but also inhibiting the development of chronic inflammation.

## 1. Introduction

Tens of millions of people suffering from bone dysfunction are estimated to require surgical intervention. As life expectancy increases, so do the number of environmental factors contributing to bone disease, and this figure is expected to increase from year to year [1]. There is still a search for new therapies and multifunctional materials designed for tissue regeneration that fulfil the requirements of contemporary medicine. 

Special attention is paid to local drug delivery systems in tissue engineering as promising tools for the treatment of infections, tissue regeneration and the fight against cancer. They have many advantages, including high drug concentrations combined with release at the target site at desired rates and prolonged therapeutic effects [2,3]. One common approach to the treatment of patients, who have suffered bone damage or are at risk of postoperative pain and rejection of the implant, is the use of non-steroidal anti-inflammatory drugs (NSAIDs) [4,5]. The benefits of these substances result from their anti-inflammatory and analgesic effects [5]. Interestingly, the pharmacodynamics of NSAIDs indicates that they affect all stages of bone and surrounding tissue healing [6]. Chang et al. reported that such drugs, i.e., celecoxib, inhibited proliferation and induced cell death in human osteoblasts (hOBs) models in concentrations, in the range of 10^−6^ to 10^−5^ M. In the case of drugs, i.e., indomethacin, ketorolac, diclofenac (DIC) and piroxicam, no significant cytotoxic effects were demonstrated for therapeutic doses (10^−5^ M) [7]. Similar reports were presented by Diaz-Rodriguez et al. who proved that an appropriate therapeutic dose of ibuprofen (<25 × 10^−3^ M) in osteosarcoma cell line culture (MG-63) does not inhibit the growth of osteoblasts and does not induce cell death [8]. 

Tissue engineering introduces the possibility to design bone substitutes in a way that allows the dosing of bioactive substances facilitating and improving the healing process. Most often bone tissue regeneration materials should be osteoinductive, osteoconductive, bioresorbable, biocompatible, easy to use, cheap and structurally similar to bone [9]. These properties can be provided by hydroxyapatite bioceramics (Ca_10_(PO_4_)_6_(OH)2—HAp), tricalcium phosphate materials (Ca₃(PO₄)₂—α,β-TCP), amorphous calcium phosphate (ACP) as well as their composites (BCP—biphasic calcium phosphates) with organic polymers [10,11]. The combination of a ceramic matrix with polymers, both synthetic and of natural origin, creates great opportunities to improve the mechanical, physicochemical and biological properties of the obtained composites. What is more, their macro and microporosity of bioceramics bone scaffold promotes the formation of features that correspond to the growth of new tissue [12]. Numerous reports on the use of polymeric systems containing NSAIDs in the regeneration of bone and cartilage tissue have appeared in the literature. An example is the biodegradable polyurethane system used in cartilaginous tissue regeneration, which provides prolonged release of DIC to 120 days [13]. Subramanian et al. produced thin flexible electrically driven membranes consisting of a copolymer of salicylic acid/succinic anhydride (SAPAE) and polycaprolactone (PCL) for use in bone tissue regeneration. These membranes were able to release salicylic acid during hydrolysis of one of the polymeric components [14]. Some studies described the effects of DIC release on reducing host response to the implanted foreign body. Sidney et al. reported the use of polymeric systems based on a copolymer of dl-lactic acid and glycolic acid (PLGA) and poly(ethylene glycol) (PEG) with DIC, which are good for the treatment of acute inflammation, thanks to the possibility of releasing 80% of the dosed active substance during the first four days [5]. Composites based on poly(anhydride ester) and ceramics (85% β-TCP and 15% HAp) were presented as targeted bone regeneration systems that are also able to reduce inflammation, thanks to the possibility of releasing salicylic acid in a hydrolysis process [15].

Polyhydroxyalkanoates (PHAs) are a group of bacterial biopolyesters, which can be synthesized by numerous bacteria under unfavourable conditions from a variety of carbon sources [16]. These macromolecules are characterised by an extended resorption time, making them an excellent organic material for the production of hybrid organic-inorganic composites for bone tissue [17,18,19]. This is possible due to the numerous advantages of these biopolymers, which include: biocompatibility, nontoxicity to human tissues and blood; bone-like piezoelectric properties; and controlled biodegradation, which is dependent on the composition of the polymer chain. 

In recent years, these compounds were the subject of interest of scientists involved in the production of materials for medical applications [17,18,19,20,21,22,23,24,25,26,27,28,29,30,31,32,33,34]. Moreover, the attention of many researchers was focused on the chemical modification of PHAs and their copolymers in order to reach a stable chemical bond between the active substance and the matrix of the polymer or oligomer. These approaches have led to the development of controlled release systems for active substances [25,28,35,36,37,38]. To the best of our knowledge, there are no reports in the literature on the synthesis of composites based on tricalcium phosphates and polyhydroxyalkanoates with covalently attached molecules of (NSAIDs). Such materials could be panacea in the regeneration of bone defects after surgical interventions caused by both tumours and other diseases, i.e., rheumatoid arthritis and osteoarthritis. The polymer layer containing a NSAID—diclofenac, would allow the delivery of the active substance directly to the implanted site. In addition, bacterial biopolymer degradation products—3-(*R*)-hydroxyalkanoic acids—nourishes the wound, reducing the risk of implant rejection. The obtained modified oligomers and their blends containing different amounts of modified oligomers were analysed by several techniques, i.e., NMR, IR, XPS, GPC, AFM, TGA and DSC. In vitro studies were carried out with the use of the MC3T3-E1. The in vitro biocompatibility studies and indirect cytotoxicity tests based on the Alamar Blue reduction were performed. Furthermore, cell proliferation and penetration into porous composites were analysed by SEM and confocal microscopy.

## 2. Materials and Methods 

### 2.1. Materials

The following chemicals were used: the octanoic acid, butyric acid, *p*-toluenosulfonic acid monohydrate (*p*-TSA), lithium hydroxide monohydrate, fetal bovine serum (FBS) calcium hydroxide and diiodomethane (Sigma Aldrich, Poznań, Poland); phosphoric acid (POCH, Gliwice, Poland); the α-MEM modification essential medium, trypsin and Alamar blue (Thermofisher, Cambridge, UK); the fetal bovine serum, pencillin, streptomycin, Dulbecco’s phosphate-buffered saline (Dulbecco’s PBS), trypan blue, dimethyl sulfoxide, ethanol and phosphate buffer saline (Sigma Aldrich, Dorset, UK), the Dulbecco’s Modified Eagle Medium (DMEM) and 4′,6-diamidino-2-phenylindole (DAPI) (Thermofisher, Warsaw, Poland); the hydrochloric acid, methanol, ethyl acetate and chloroform (Chempur, Poland); the sodium chloride and the tetrahydrofuran chromatographic grade (Avantor Chemicals, Gdańsk, Poland); the methanol and acetonitrile HPLC grade (Chemsolve, Łódź, Poland), and the diclofenac acid (Fluorochem, Hadfield, UK).

### 2.2. Preparation of the Investigated Materials

#### 2.2.1. Production of Poly(3-hydroxyoctanoate) (P(3HO)) Polymer

P(3HO) polymer, which was composed of 91 mol.% (*R*)-3-hydroxyoctanoic acid, 7 mol.% (*R*)-3-hydroxyhexanoic acid and below 2 mol.% in total of other (*R*)-3-hydroxylated fatty acids, was obtained in the process of controlled feeding of the *Pseudomonas putida* KT2440 [39] strain in a 5 L fermenter. Octanoic acid was used as the sole energy and carbon source. A detailed process of its production and characterization were described previously [21].

#### 2.2.2. Preparation of Highly Porous Tricalcium Phosphate (TCP) Ceramics

The initial TCP powder was synthesised via a wet chemical method according to a procedure described previously [17]. The calcium hydroxide (Ca(OH)_2_, ≥99.5%) and phosphoric acid (H_3_PO_4_, 85.0%) were applied as reagents. The obtained precipitate was dried, calcined at 900 °C and grounded in attritor into a grain size below 0.063 mm. Ceramic slurry with a high amount of β-TCP powder was used to obtain highly porous bioceramics scaffolds via the polyurethane sponge replica method. 

#### 2.2.3. Preparation of Oligo-DIC Conjugates (Fin-Dic-oliP(3HO))

In order to prepare oligo-DIC polymer conjugates (Fin-Dic-oliP(3HO)), modified protocol described by Kwiecień et al. was used [35]. For this purpose, 5 g of P(3HO) polymer, 2 g (40% *w*/*w*) of DIC and 0.75 g (15% *w*/*w*) of *p*-TSA were introduced into a round bottom flask equipped with a magnetic stirring bar. The reaction was performed at 125 °C under an argon atmosphere, and started when the molten state of all reagents was observed. The reaction time was 2 min, after which the mixture was cooled in an ice bath and 50 mL of chloroform was added. The organic layer was washed five times with 1 M sodium chloride aqueous solution and two times with water to remove residual p-TSA. Then, the solvent was evaporated under vacuum. The reaction was performed in triplicate. 

#### 2.2.4. Preparation of Ceramic-Polymer Composites

To obtain polymeric coatings, three blends of P(3HO) with drug-modified oligomers were prepared: two (2Dic-oliP(3HO)), one (1Dic-oliP(3HO)) and a half (0.5Dic-oliP(3HO)) of a therapeutic DIC dose [40] (1 dose = 0.19 g DIC per 1 g of polymer) (Table 1). In this order, an appropriate amount of P(3HO) was dissolved in 50 mL of dichloromethane at 60 °C, which followed the addition of an appropriate amount of oligo-DIC [41]. The constituents were mixed for 3 h at an ambient temperature after the solvent was evaporated. The blends were dried in a vacuum dryer for 24 h, then dissolved in ethyl acetate in the concentration of 5% *w*/*v* and used to infiltrate the ceramic scaffolds [17].

### 2.3. Physicochemical Analysis of Oligo-DIC Conjugates and Their Blends

#### 2.3.1. Nuclear Magnetic Resonance (1H NMR) Analysis

^1^H NMR spectra were obtained in CDCl_3_ with tetramethylsilane (TMS) as the internal standard using a 300 Hz spectrometer (Bruker BioSpin GMbH, Rheinstetten, Germany). Number of scans: 56; pulse width: 14.5 s; acquisition time: 3.5 s.

#### 2.3.2. Attenuated Total Reflection Fourier Transform Infrared (ATR-FTIR) Analysis

FTIR spectra were recorded using a Nicolet iS5 spectrometer (Thermo Fisher Scientific, Waltham, MA, USA) equipped with a diamond crystal attenuated total reflectance unit (ATR) with a resolution of 8 cm^−1^ from 4000 cm^−1^ to 400 cm^−1^ as well as with an average of 16 scans.

#### 2.3.3. Gel Permeation Chromatography (GPC) Analysis

Molecular weight distributions of oligo-DIC and oligo-DIC/P(3HO) blends were analyzed using modified protocol described by Sofińska et al. [21] Spectroscopic-grade tetrahydrofuran was used as an eluent at a flow rate of 1.0 mL min^−1^. Sample concentration was 30 g L^−1^, whereas injection volumes were 50 µL. All measurements were performed in triplicate.

#### 2.3.4. Thermal Analysis

Thermogravimetric analysis (TGA) was carried out using a Netzsch STA 409 PC Luxx according to protocols described by Haraźna et al. [42] Differential scanning calorimetry (DSC) experiment analyses were performed with a Mettler Toledo 822e calorimeter purged with argon. The samples were first heated from room temperature to 200 °C at 10 K min^−1^ to erase the thermal history, then the samples were cooled at 10 K min^−1^ to −80 °C and second heating run at 10 K min^−1^ were accomplished.

#### 2.3.5. X-ray Photoelectron Spectroscopy (XPS)

X-ray photoelectron spectroscopy (XPS) was applied to determine the surface composition and surface interaction of P(3HO) with DIC deposited on a silicon substrate. For the analysis, the XPS spectrometer with hemi-spherical analyzer SES R4000 (Gammadata Scienta, Uppsala, Sweden) and a Mg Kα radiation source (1253.6 eV) was used. The energy resolution of the spectrometer was equal to 0.9 eV (Ag 3d5/2 at pass energy of 100 eV). The spectrometer was calibrated according to ISO 15472:2010. A thin layer sample was placed on a dedicated holder, after the holder was pumped (8 h) to a high vacuum and then introduced into the UHV. The analysis area was 3 mm2, the spectra were deconvoluted with CasaXPS 2.3.15. 

#### 2.3.6. Measurements of the Contact Angle and Determination of Surface Free Energy

Thin films were prepared by a solvent casting technique. The Fin-Dic-oliP(3HO) and its blends were dissolved in chloroform at 10% *w*/*v* concentration and 100 µL of prepared solutions were deposited twice onto a glass plate surface and such prepared films were left at room temperature at normal pressure for 2 weeks. The wetting angles were obtained with Drop Shape Analyzer KRUSS DSA100M optical contact angle measuring instrument (Kruss Gmbh, Hamburg, Germany). The surface free energies were determined using an Owens-Wendt (OW) method. The polar and dispersive components were calculated.

#### 2.3.7. Atomic Force Microscopy (AFM) Imaging

The samples for AFM study were prepared on glass support. Briefly, the blends were dissolved in ethyl acetate at 5% *w*/*v* concentration. On a glass surface of plates an aliquoted 50 µL of prepared solutions were dispensed and dried in a dryer at 50 °C for 15 min. After that the plates were kept at room temperature, normal pressure for 1 week. AFM images of the prepared films were recorded with a Veeco Innova atomic force microscope in a tapping mode, with tips of resonant frequency of ca. 300 kHz. Images used for further evaluation were prepared in canvases of 10 × 10 μm, with resolution of 512 × 512 pixels (for 0.5Dic-oliP(3HO) and 1 Dic-oliP(3HO)) or 256 × 256 pixels (for 2 Dic-oliP(3HO)). Images were levelled by subtraction of a fitted plane. Minor mutual shifts between line profiles along the fast-scanning axis, due to instrument noise, were smoothed by subtraction of the mean of each line. Root mean square (RMS) roughness (*Sq*) was calculated on the corrected images as:

(1) $$S_{q}=\sqrt{\frac{1}{N}\overset{N}{\underset{n=1}{\sum }}{\left(z_{n}-\bar{z}\right)}^{2}}$$

where $z_{n}$ is the height at point n and $\bar{z}$ the mean height [43].

All mathematical operations on the AFM images were conducted with the Gwyddion software [44].

#### 2.3.8. SEM Imaging

Scaffolds were sputtered with gold and analysed with the JEOL JSM—7500F Field Emission Scanning Electron Microscope equipped with Retractable Backscattered-Electron detector (RBEI) and EDS (energy dispersive spectra) detection system of characteristic X-ray radiation INCA PentaFetx3 EDS system (JEOL Ltd., Tokyo, Japan). 

#### 2.3.9. Confocal Imaging

Confocal images were obtained using Zeiss Axio Observer Z.1 microscope with LSM 710 confocal module. Image postprocessing was performed in dedicated software using Zeiss ZEN Black version 8,1,0,484, PALMRobo V 4.6.0.4 and FluoRender 2.21.0. Observations were made with an air 10× objective. For DAPI stain excitement a wave with length of 405 nm was applied [45].

### 2.4. Biological Characterisation of Prepared Ceramic-Polymer-Scaffold Composites

#### 2.4.1. The Mouse Calvaria-Derived Pre-Osteoblastic (MC3T3-E1) Cell Culture

MC3T3-E1 cells (CRL-2593, ATCC, London, UK) were cultured in standard tissue culture flasks in α-MEM supplemented with 10% vol. FBS and 5% vol. penicillin/streptomycin antibiotics and maintained at 37 °C in a humidified atmosphere of 5% CO_2_. The media were replaced every 2 days. When 80% confluency was reached, cells were washed twice with Dulbecco’s PBS and detached using 0.25% trypsin containing 1 mM EDTA. After centrifuging the solution (5 min, 1500 rpm), the cells were suspended in α-MEM, and the number of cells was counted using Neubauer chamber. The cells were either seeded on the scaffolds or cultured again in a standard tissue flask.

#### 2.4.2. The Cytotoxicity Test of Scaffold Extracts (Indirect Cytotoxicity)

The indirect cytotoxicity studies were carried out to evaluate the presence of toxic compounds in the extracts obtained from the scaffolds, following the ISO 10993-5, using a protocol described by Skibiński et al. [41].

#### 2.4.3. In Vitro Biocompatibility Studies

Each scaffold was sterilised with ethylene oxide at 37 °C for 12 h and placed into a well of a 24-well tissue culture polystyrene plate. After this, the scaffolds were pre-wetted with 1.7 mL α-MEM for 1 day. The 24-well plates were incubated at 37 °C in a humidified atmosphere of 5% CO_2_. Next, for attachment and proliferation studies, the cells were seeded directly on the scaffolds at a density of approximately 60,000 cells per well. The positive control consisted of cells cultured directly on tissue culture plastic with 1 mL of α-MEM medium. For the proliferation study, after 1, 3 and 7 days of culturing, the viability of cells for each scaffold was determined using the Alamar Blue assay (note: plates incubated for 7 days had medium replaced on the 3rd day).

#### 2.4.4. Alamar Blue Assay

The metabolic activity of the cultured cells was measured using Alamar Blue. To determine the viability of cells, each cell culture medium was replaced with an appropriate amount of 10% *v*/*v* Alamar Blue in α-MEM medium (i.e., for direct tests—1.7 mL and indirect tests—150 µL) and incubated at 37 °C in a humidified atmosphere of 5% CO_2_ for 3 h. After this, 100 µL of each solution was transferred to 96 well plates and absorbance at 570 nm and 600 nm was measured on a plate reader. The absorbance of the samples was normalised concerning the positive control.

#### 2.4.5. Cell Morphology Observations

Specimens obtained from direct cytotoxicity assays were fixed twice by soaking in a Dulbecco’s PBS, followed by a treatment with 4% *v*/*v* formaldehyde PBS solution and then stored at 4 °C. Next, the cells were gradually dehydrated by immersing in 2 × PBS solution and ethanol aqueous solutions of 35%, 50%, 70%, 90% and 2× in 98% *v*/*v*; each for 15 min. The analyses of cell attachment, colonization and microstructure of the scaffolds were performed using scanning electron and confocal microscopies. 

### 2.5. Statistical Analysis

The results were expressed as mean values ± standard error (SE). The analysis of variance (ANOVA) was performed among groups for two population comparisons. The differences were deemed statistically significant at probabilities of * *p* < 0.05, ** *p* < 0.01, *** *p* < 0.001 (Origin Pro 2019 Software).

## 3. Results

### 3.1. Spectroscopic Insights into Structure of Obtained Drug-oligoP(3HO) Conjugates and Their Blends 

**Figure 1:**
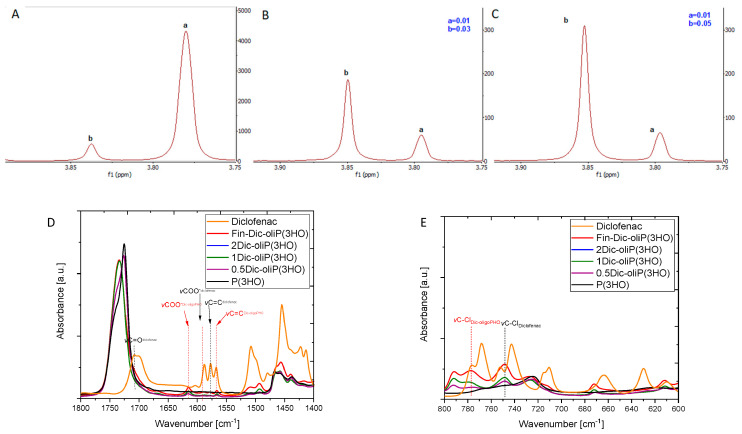
(**A**). ^1^H NMR of Fin-Dic-oliP(3HO) analysis. (**B**). ^1^H NMR analysis of the working sample before the addition of 4 drops of DIC solution (25 g L^−1^). (**C**). ^1^H NMR analysis of the working sample after addition of 4 drops of DIC acid solution (25 g L^−1^), where (a) denotes the CH_2_ groups from DIC attached to oligomers via an ester bond, (b) groups from free DIC. (**D**). Infrared spectra of the obtained compounds zoom in between 1800–1400 cm^−1^. (**E**). Infrared spectra of the obtained compounds zoom in between 800–600 cm^−1^ ranges**.**

The ^1^H NMR spectra analyses (Figures S1 and S2) reveal the presence of signals that are assigned to protons originating from the 3-hydroxyoctanoic acid repetitive unit and 10-fold less intense signals originating from the 3-hydroxyhexanoic acid. One should stress that it is possible to distinguish between chemically bonded DIC to the oligomers via an ester bond (Figure 1A, signal a) and the remaining, unreacted and physically mixed compound (Figure 1A, signal b). This is confirmed by adding pure DIC to the post-synthesis sample and observing the change in the signal intensity assigned to physically mixed DIC at 3.85 ppm (Figure 1B,C, see the increase of peak b). 

**Figure 2:**
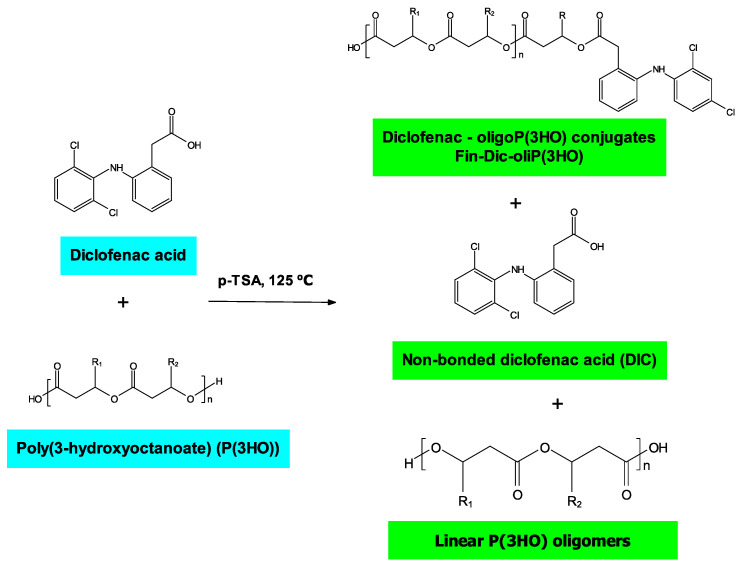
Species formed during the reaction of P(3HO) with DIC catalyzed by *p*-TSA.

What is more, we have shown that linear oligomers without the attached modifier are also present in the mixture (Figures S1 and S2). Additionally, the high temperature of the reaction results in the formation of unmodified oligomers terminated with unsaturated end groups. Moreover, we have determined that 89% of DIC is attached to PHA oligomers and 11% remains free. The structure of the compounds included in the whole obtained mixture is presented in Figure 2. The formation of analogous structures as we present them here was also observed in other works [35,36]. 

From Figure S3 it is seen that the spectra of the final product (Fin-Dic-oliP(3HO)) and the resulting blends with the P(3HO) polymers (2Dic-oliP(3HO), 1Dic-oliP(3HO) and 0.5Dic-oliP(3HO)) contain vibrations characteristic mainly for the P(3HO) polymer as well as new vibrations and small changes compared with the drug molecules (Figure S3). The bands at 3000–2800 cm^−1^ are attributed to the vibrations of methylene (CH_2_) and methyl (CH_3_) groups, whereas the peak at 1732 cm^−1^ is attributed to the stretching vibrations of the carbonyl (C=O) group of the P(3HO) polymer [21,46]. The obtained DIC spectrum is similar to the one reported in the literature [47]. The visible absorption peaks at 1710 cm^−1^ and 1589 cm^−1^ indicated stretching vibrations of C=O and COO^−^ groups. The stretching vibrations in the aromatic ring of C=C groups are observed at 1579 cm^−1^. The vibrations of secondary amine groups are visible due to stretching of N-H and C-N groups at 3324 cm^−1^, 1284 cm^−1^ and 1162 cm^−1^. Additionally, the band at 752 cm^−1^ is attributed to the stretching vibrations of C-Cl groups. The visible change in the shape of the bands corresponding to the vibrations of the C=O groups at 1732 cm^−1^ is a premise confirming the formation of an ester bond between the drug molecule and the polymer (in Fin-Dic-oliP(3HO), Figure 1D). A similar phenomenon was observed by Gumel et al., who confirmed the successful modification of polymer from the mcl-PHA group by a biocatalytic approach using sucrose as a modifier [48]. Furthermore, the changes in the shape of the same bands are also observed in the prepared blends (2Dic-oliP(3HO), 1Dic-oliP(3HO)). Moreover, due to the low content of Fin-Dic-oliP(3HO) in the 0.5Dic-oliP(3HO) blend, the shape of the C=O bands did not differ much from the one of P(3HO). Modification of oligomers by DIC is also confirmed by the presence of the band characteristic for C-Cl stretching vibrations at 792 cm^−1^ (Figure 1E) and C=C and COO^−^ stretching vibrations at 1565 cm^−1^ and 1615 cm^−1^ (Figure 1D). Additionally, bands visible at 1589^−1^ and 1579 cm^−1^, which indicate vibrations of C=O and C=C groups of DIC, substantiate the presence of unbound DIC in the prepared samples. 

**Figure 3:**
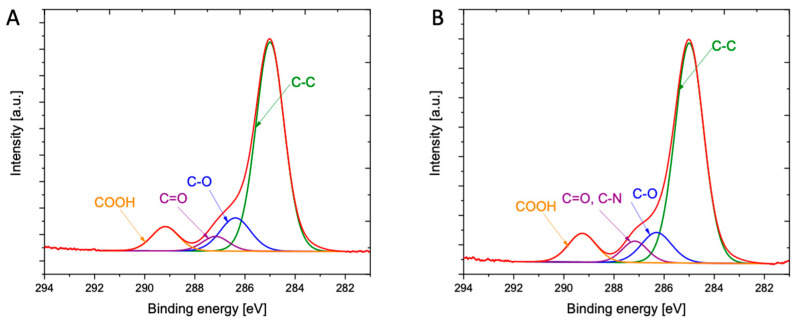
XPS analysis of deconvoluted C 1s spectra, (**A**) shows P(3HO) spectrum and (**B**) indicates Fin-Dic-oliP(3HO) spectrum.

Results of XPS used to analyze the elemental composition of investigated materials are summarized in Figure 3 and Figure S4. The modified polymeric oligomers (Fin-Dic-oliP(3HO)) exhibit two new peaks (N 1s and Cl 2p), indicating the presence of DIC molecules. Moreover, the O/C ratio decreases with the addition of modified oligomers. As shown in Table 2, the surface of the P(3HO) polymer is composed of 77.25% of carbon and 22.75% of oxygen, whereas the surface of modified oligomers (Fin-Dic-oliP(3HO)) contain 79.27% of carbon, 20.11% of oxygen, 0.46% of nitrogen and 0.17% of chlorine. For P(3HO), the O 1s spectrum consists of two dominant peaks at 532.2 eV and 533.4 eV due to its presence in (C=O)-O* and (C=O*)-O-groups, respectively, and a smaller one at 534.4 eV assigned to oxygen in terminal OH groups in the P(3HO) chain (Figure S5A and Table S1). There is no significant difference observed for the modified oligomers with regards to O 1s (Figure S5B). However, when C 1s spectra for the P(3HO) and modified oligomers are compared, increased content of carbon atoms corresponding to C-O and C=O groups confirms the formation of an ester bond between oligomers and DIC (Figure 3A,B, Table S2). Liu with co-workers observed a similar phenomenon after cross-linking gentamicin with chitosan, where C-N, C-O and C=O peak intensity increased after modification [49].

### 3.2. Physicochemical Characteristics of Fin-Dic-oligoP(3HO) Conjugates and Their Blends

It is known that the molecular weight of the polymer plays an important role in the context of carrier degradation and release of the active substance. We have found that the modification of P(3HO) using DIC yields compounds with an average molecular weight of 4.92 kDa, whereas the dispersity index (DI) for P(3HO) and Fin-Dic-oliP(3HO) are 2.19 and 1.66, respectively (Table 3).

**Figure 4:**
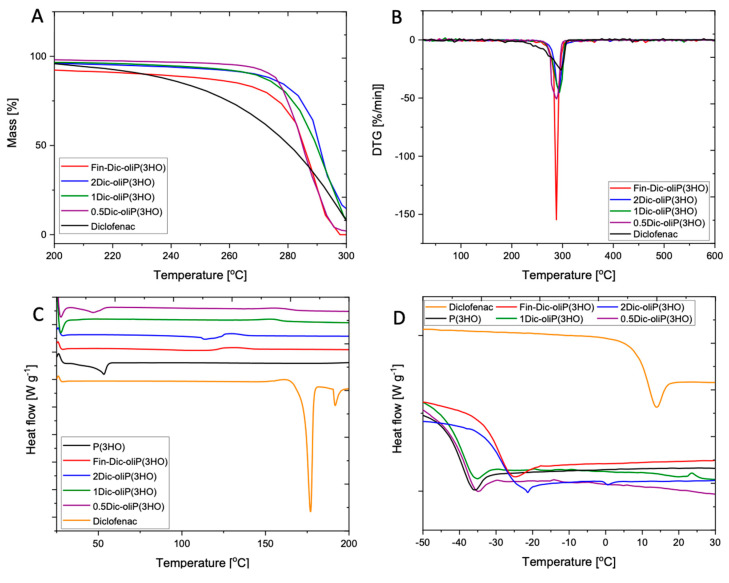
(**A**,**B**) Thermogravimetric (TGA); (**C**,**D**) Differential Scanning Calorimetry (DSC) results of the materials used in this study.

Figure 4A,B shows changes in the processes related to the initiation of thermal degradation of the basic materials, i.e., DIC, P(3HO), modified oligomers (Fin-Dic-oliP(3HO) as well as blends prepared using 2Dic-oliP(3HO), 1Dic-oliP(3HO) and 0.5Dic-oliP(3HO). In the case of inert thermal degradation, a one-stage process of material degradation is observed in all analyses (Figure S6). The reverse dependence was observed for the products of a high molecular weight medium chain length polyhydroxyalkanoates (mcl-PHAs) modification with sucrose as well as copolymer of 3-hydroxybutyric acid and 3-hydroxyvaleric acid (P(3HB)-co-(3HV)) with ascorbic acid—in both cases the degradation processes were two-staged [48,50]. Moreover, our analyses indicate a slight increase in temperature at which degradation started, in case of modified oligomers (Fin-Dic-oliP(3HO)) as well as blends containing two doses of active substance per 1 g of polymer (2Dic-oliP(3HO)). The onset of thermal decomposition processes (T_onset_) of 1Dic-oliP(3HO) and 0.5Dic-oliP(3HO) blends show no significant differences (Figure 4A,B, Table 3).

DIC is a strongly crystalline substance, showing two sharp melting endotherms at 177 °C and 192 °C (Figure 4C, Table 3). This crystallinity is not preserved when modified with the P(3HO) oligomer. P(3HO) curves show a weak and broad melting peak at 53 °C. Crystallinity is preserved when blended with a small amount of the modified DIC (sample 0.5Dic-oliP(3HO)), albeit the melting temperature (T_m_) drops by a few degrees, indicating reduced quality of the crystals. However, more modified DIC completely disrupts the development of crystalline order as manifested by the absence of melting endotherms in the other blends. Glass transition temperature (T_g_) of neat P(3HO) was observed at 41 °C (Figure 4D, Table 3). Pure DIC has a much higher T_g_ at 10 °C. Their compound (Fin-Dic-oliP(3HO)) has an intermediate T_g_ closer to that of the oligomeric decorations, as expected in the sense of mixing laws, such as that of Fox [51]. The 0.5Dic-oliP(3HO) and 1Dic-oliP(3HO) blends have T_g_ practically equal to pure P(3HO), while the mass fraction of Fin-Dic-oliP(3HO) in the blend is significant (24% *w*/*w* and 48% *w*/*w*; Table 3). This indicates a possible phase separation between the two components, despite the fact that no second glass transition is observed. Indeed, the AFM observations (Section 3.4) showed the development of Fin-Dic-oliP(3HO)-rich nano regions. On the contrary, the sample 2Dic-oliP(3HO) with a mass fraction of Fin-Dic-oliP(3HO) (96% *w*/*w)* has T_g_ practically equal to that of its majority component, as expected in the sense of mixing laws.

### 3.3. Wettability and Surface Free Energy Determination

**Figure 5:**
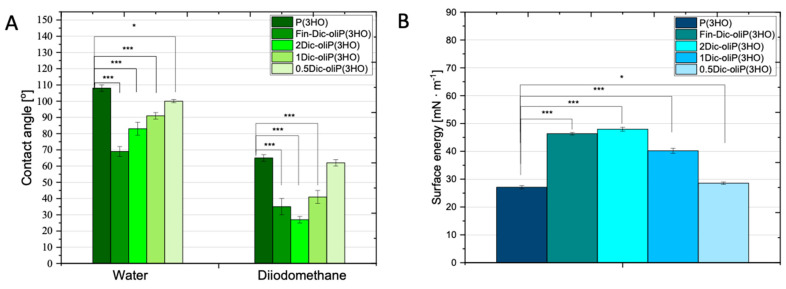
(**A**) Wettability characteristics of polymeric/oligomeric films in contact with water and diiodomethane (*n* = 6; error bars = ±SD). (**B**) Surface free energy of materials (*n* = 36; error bars = ±SD). The results are statistically significant, where: * *p* < 0.05, *** *p* < 0.001.

In order to determine the wettability and surface energy of both the unmodified polymer and the obtained blends, we have measured the wettability angle for a polar liquid (water) and non-polar diiodomethane. One should stress that according to the literature data, concerning biomaterial analysis, hydrophobic properties of a given material are considered when wetting angle values are above 70° [32]. In our experiment, we observed a decrease in hydrophobicity of the material with modification of P(3HO) oligomers and wetting angle values for unmodified polymer and modified oligomers equal to 106 ± 2° and 69 ± 3°, respectively (Figure 5A). In other works, Lukasiewicz et al. demonstrated that the increase in the number of medium-chain oligomers (mcl-oliPHA) caused an increase in the hydrophilicity of the material [31]. In this case, for the initial material poly(3-hydroxybutyrate) P(3HB) and its blend (0.8 P(3HB)/0.2 mcl-oliPHA), wettability was 87.5 ± 7.4° and 118.0 ± 7.0°, respectively [31]. In our research, DIC modified oligomers and blends obtained from them have a wettability for water and diiodomethane of the values: for Fin-Dic-oliP(3HO) (69 ± 3°, 35 ± 5°), 2Dic-oliP(3HO) (74 ± 2°, 27 ± 3°), 1Dic-oliP(3HO) (91 ± 2°, 41 ± 4°) and 0.5Dic-oliP(3HO) (100 ± 1°, 62 ± 2°). Similar wettability for the unmodified polymer P(3HO) was determined by Cichoń et al., i.e., 100 ± 6° for water and 52 ± 3° for diiodomethane [17].

The analysis of surface free energy done by us revealed the similarity of the values obtained for both unmodified polymer and 0.5Dic-oliP(3HO)-coated composite. The values of the polar, dispersion and total free energy components are 0.38 ± 0.04, 27.71 ± 0.30, 27.06 ± 0.40 mN m^−1^ and 0.84 ± 0.09, 27.7 ± 0.44, 28.55 ± 0.42 mN m^−1^, respectively, for the unmodified and modified materials (Figure 5b and Figure S7A,B). Strict dependence is also observed for the polar component and total surface energy. With increasing concentration of DIC modified oligomers in prepared blends both polar components and free surface energy increased. No biased relation is seen for the dispersion component. The values for P(3HO), 0.5Dic-oliP(3HO), 1Dic-oliP(3HO), 2Dic-oliP(3HO) and Fin-Dic-oliP(3HO) are 27.71 ± 0.30, 27.71 ± 044, 38.67 ± 0.79, 40.67 ± 0.45 and 35.14 ± 0.74 mN m^−1^, respectively.

### 3.4. Surface Roughness Determination

**Figure 6:**
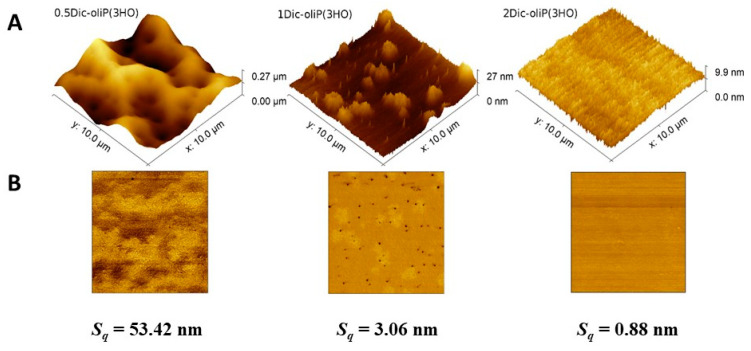
AFM images of Fin-dic-oliP(3HO)-based blend film (10 × 10 μm^2^). (**A**): The height images in 3D representation Note the different scales on the *z*-axis. (**B**): The corresponding phase images.

Due to the fact that the surface of the materials may affect the activity of cells, allowing them to grow in a specific direction, it is necessary to determine the topographic parameters of the prepared blends [52]. Similar to the research described by Sofińska et al. of the surface of a non-modified P(3HO) polymer, all AFM images indicate the presence of micro- and nano-scale structures with different roughness [21]. Moreover, AFM reveals an impressive change in the morphology of the surface of the blends under investigation, as seen in the height images in Figure 6. The 0.5Dic-oliP(3HO) blend shows a rough topography, however, with characteristic microcavities. A similar surface topography for Poly(l-lactic acid) (PLLA)/Polystyrene (PS) composition was observed by Lim et al. [53]. The resulting microcavities can influence the cell activity in the regenerated tissue [52]. 

In the other extremum, 2Dic-oliP(3HO) shows no features at all. In this blend, Fin-Dic-oliP(3HO) is the majority component (96% *w*/*w*) and presumably P(3HO) is fully miscible in it, creating a fully homogeneous material, as manifested in both the topographic and phase images. In the intermediate case of 1Dic-oliP(3HO), topography shows features of two types distributed in a flat background. The two features are globules of a radius in the order of 1 μm, and particles of a radius in the order of 100 nm. The corresponding phase image reveals that the two types of features have different mechanical properties. On a closer look, the smaller structures are visible to a much lesser extent in the phase image of 0.5Dic-oliP(3HO). We assume thus that they correspond to Fin-Dic-oliP(3HO)—rich areas where the phase separates from the polymer. This observation is in agreement with the observations about their T_g_ in Section 3.2. 

Additionally, the root mean square roughness (*S_q_)* of the samples surfaces was determined (Figure 6). A decreasing value of this parameter for the three prepared blends with an increasing content of Fin-dic-oliP(3HO) was observed. A similar phenomenon was described in the case of formulations based on polyvinyl alcohol and boric acid (PVAB) with DIC sodium salt [54]. Taking into account the fact that the 0.5Dic-oliP(3HO) blend consists of ca. 75% *w*/*w* of the P(3HO) polymer, the *S_q_* is significantly higher (53.42 nm) compared to the research carried out by Sofińska et al. (13.2 ± 2.8 nm) on pure P(3HO) [21]. This may be due to the addition of Fin-dic-oliP(3HO), which acts as a co-crystallizer, reducing the polymer structure, thus allowing the formation of microcavities [55].

### 3.5. Cytotoxicity of Dic-oliP(3HO) Conjugates-Based Blends

**Figure 7:**
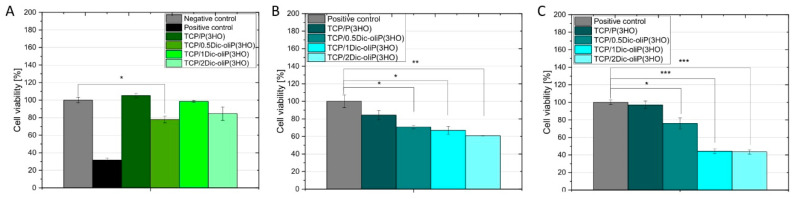
(**A**). Indirect cytotoxicity test showing no positive impact of the 1-day old leachate on MC3T3-E1 mouse pre-osteoblasts (*n* = 3; error bars = ±SD). In vitro biocompatibility study results of cells seeded on the materials on day 1 of the experiment (**B**) versus day 7 (**C**) (*n* = 3; error bars = ±SD). Besides the TCP/P(3HO) composite, only theTCP/0.5Dic-oliP(3HO) scaffold allowed for 76.0 ± 6.4% cell viability after 7 days of incubation, thus making it non-cytotoxic according to ISO 10993-5. The results are statistically significant, where: * *p* < 0.05, ** *p* < 0.01, *** *p* < 0.001.

Cytotoxicity of DIC-modified oligomers and prepared blends are evaluated in the indirect cytotoxicity test, in which the exudate from material incubation in the cellular medium is analyzed (Figure 7A). Typical sources of cytotoxicity are residues of organic solvent from polymer synthesis, intermediate products used for synthesis and material degradation products as well as the additives themselves with which the polymer is modified [56]. Figure 7 summarizes indirect cytotoxicity studies and in vitro biocompatibility tests performed using an MC3T3-E1 model to assess the possibility of using the prepared bioceramic-polymer composites in bone tissue regeneration processes. According to ISO 10993-5, a 30% reduction in cell viability may indicate cytotoxicity of the material [57]. All studied material extracts show no cytotoxic effects. What is more, higher cell viability is achieved for the unmodified composites (105 ± 3%) compared to the negative control, which confirms that neither β-TCP nor P(3HO) contain or release harmful components for the MC3T3-E1 cell line. This substantiates the excellent biocompatibility of the composites components—according to the literature, both PHAs and TCPs show no cytotoxicity to human tissues [10,24]. Additionally, the release of polymer decomposition products—(*R*)-3-hydroxyacids—stimulates cell viability [58]. The lack of cytotoxicity of materials made of β-TCP is confirmed by the results obtained by Skibiński et al. Indirect cytotoxicity tests of β-TCP sponges performed in the MC3T3-E1 model showed high cell viability (117.11 ± 5.59%) [41]. In the case of composites infiltrated with blends containing different amounts of Fin-Dic-oliP(3HO), no cytotoxic effects with respect to the negative control are observed. For TCP/0.5Dic-oliP(3HO), TCP/1Dic-oliP(3HO) and TCP/2Dic-oliP(3HO) cell viability is equal to 78 ± 4%, 99 ± 1% and 85 ± 8%, respectively. From the analysis, it can be concluded that the amount of active substance released during the 24 h of incubation does not cause a cytotoxic effect when in contact with the MC3T3-E1 cell line. In the case of β-TCP/P(3HO) composites containing physically bounded DIC, analogous phenomenon was reported (99.20 ± 5.61%) [41]. Moreover, a similar effect was observed by Sidney et al. after a 14-day incubation of the mouse pre-osteoblast cells in the culture medium and further addition of 1 mg of sodium DIC per scaffold (8-fold lower than that used in our study) [5].

### 3.6. In Vitro Biocompatibility and Cell Morphology on Hybrid Composites

The selection of an appropriate preparation method and the proper composition of the scaffold allows us to obtain the best possible biochemical and mechanical properties, which may lead to increased adhesion, differentiation and proliferation of the mammalian cells; it may simulate the environment around natural tissues. An important feature of the scaffold, apart from its porous structure, is its bioabsorption and biocompatibility [31,32,33,34]. For these reasons, we have carried out direct compatibility analysis, seeding the (MC3T3-E1) directly on our materials (Figure 7B,C). Cell proliferation ability on surfaces based on PHAs was evaluated already in various cell models [25,26,27,31,32,33,34,59]. Our study shows increased viability along with the time of cell incubation on the surface of TCP/P(3HO) composites. After 1 and 7 days of incubation, the cell viability for TCP/P(3HO) composites are 84.3 ± 5.3% and 97.1 ± 4.4%, respectively, compared to the negative control. Taking into consideration the composites containing chemically bonded DIC, the cell viability at day 1 and 7 changes as follows: for 0.5Dic-oliP(3HO) from 71.0 ± 1.9% to 76.0 ± 6.4%; for 1Dic-oliP(3HO) from 66.8 ± 4.5% to 44.2 ± 2.7%; and for 2Dic-oliP(3HO), from 60.9 ± 0.1% to 43.6 ± 2.4%. From the above data, we can conclude that the composite blends with the lowest dose of chemically bonded DIC (0.5Dic-oliP(3HO)) were able to facilitate the attachment and growth of MC3T3-E1 cells. For the composites with higher amount of chemically bonded DIC (1Dic-oliP(3HO) and 2Dic-oliP(3HO)), at day 1 the cell viability was comparable to 0.5Dic-oliP(3HO) samples. However, when the materials were incubated for a longer time, cell growth inhibition was detected.

**Figure 8:**
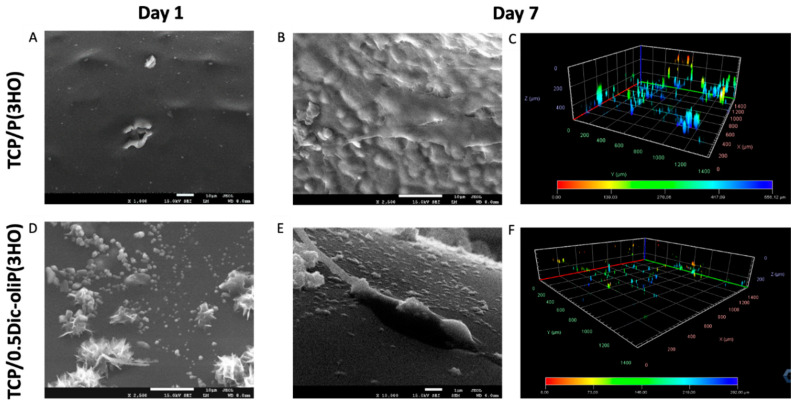
MC3T3-E1 cell colonization of TCP/P(3HO) and TCP/0.5Dic-oliP(3HO) composites. Materials on the 1st day (**A**,**D**) and on the 7th day (**B**,**E**) of incubation visualised using SEM. Bars in A,B and D correspond to 10 µm, whereas in E to 1 µm. (**C**,**F**): 3D reconstructions of cells migrating into the materials after 7-day incubation. Colour-coded depth penetration in C from 0 (red) to 556.12 µm (blue) and in F from 0 (red) to 292.0 µm (blue).

Using SEM and confocal microscopy observations we have analyzed the pre-osteoblast mouse cell colonization over time on the studied materials. Microphotographs of TCP/P(3HO) scaffolds show the formation of a uniform cell layer covering the surface of the material after 7 days, indicating excellent cell viability (Figure 8B). Similar observations were shown by Lukasiewicz et al. in the (P(3HB))/mcl-(P(3HA)) blend study with the mouse myoblast (C2C12) cell line [31]. Our scanning electron and confocal microscopy analyses of 0.5Dic-oliP(3HO) material reveal single cells and fibers originating from their presence on the composite surface, however, with lower confluency compared to unmodified scaffolds (Figure 8E). Moreover, 3D reconstructions of confocal microscopy images suggest that cells seeded on TCP/0.5Dic-oliP(3HO) migrate slower and penetrate the material less invasively than TCP composites with pure P(3HO) (Figure 8C,F). In the case of the unmodified scaffold, the cells penetrate ~500 µm deep into the material, while in the case of TCP/0.5Dic-oliP(3HO), the migration range is reduced to a maximum of ~300 µm. Analyses of the other two composites (1Dic-oliP(3HO) and 2Dic-oliP(3HO)) do not show the presence of the cells in the middle of the scaffold’s pores (Figure S9). However, their microphotographs reveal that chemical modification results in the crystallization of Fin-dic-oliP(3HO) in a different form than that for pure DIC. Fini et al. demonstrated that DIC sodium salt has the form of spindle-shaped crystals, while our research shows that modification of low molecular weight PHA oligomers with DIC results in the formation of hedgehog-like, crystalline structures [60] (Figure 8D).

## 4. Discussion

The process of bone tissue regeneration requires the use of materials with osteoinductive and osteoconductive properties, which can simulate conditions occurring in natural bone. Moreover, after the implantation, inflammation is often observed, which can be eliminated by using appropriate bioactive agents [3]. In order to obtain a polymeric delivery system containing a non-steroidal, anti-inflammatory drug namely diclofenac, we presented a single-stage, solvent-free synthesis method. Its clue relies on a simultaneous reaction of polymer, hydrolyzing agent (aimed at transforming the polymer into oligomers) and a bioactive substance (DIC-modifier) and leads to new bioactive compounds—oligomers combined with DIC. From the literature it is known that the use of *p*-TSA is one of the methods enabling the simultaneous decomposition of the polymer as well as its modification [35,36]. Other methods supporting modification and partial decomposition of the polymer is transesterification reaction using a biocatalytic approach [48]. The methods resulting exclusively in the degradation of the polymer chain comprise acidic methanolysis, alkaline saponification or thermal degradation [31]. Kwiecień et al. demonstrated that a higher temperature and a higher amount of hydrolysing agent used led to oligomers with lower molecular weight [35,36]. Lukasiewicz et al. substantiated that the reactions in which smaller molecular weights are obtained have a higher homogeneity, characterised by lower DI, which is consistent with the results obtained in [31].

In the literature there is a correlation between the average molecular weight of the polymer and the release of the active substance. Furthermore, it was shown that with a decrease in the average molecular weight, the release rate of the active substance increased [61]. Other important parameters influencing the biodistribution and absorption of characterising the active substance are those of a given surface, i.e., hydrophilicity or hydrophobicity, surface charge [62]. As it is known, immediately after the implantation, an inflammation is observed which may even lead to the rejection of the newly inserted implant [63]. Therefore, it is important to use active substance carriers, which can release more drug molecules in the first days of application and then gradually release the residue, preventing possible side effects. In addition to the release of the active substance, degradation of the polymer carrier was shown, leading to a decrease in molecular weight and a change in dispersity [61]. The use of a low molecular weight matrix is also beneficial in terms of increasing the degradation rate of the polymeric material as shown in [64]. Rajaratanam et al. reported that the use of (P(3HB)-co-(3HHx)) oligoesters favours the process of the degradation of threads made of these materials [27].

Studies conducted by Witko et al. have shown that virgin P(3HO) is a non-toxic material in contact with a mouse embryonic fibroblast cell line (MEF3T3). Additionally, it was observed that this polymer affects cell morphology, tubular cytoskeleton and cytoskeletal components, i.e., actin. The authors reported that the surface properties of P(3HO), i.e., the coefficient of friction, combined with lower brittleness of the materials compared to other biodegradable and biocompatible polyesters such as poly(lactic acid) (PLA) and P(3HB), may indicate potential applications of P(3HO) in the construction of medical implants, joint endoprostheses. It should be underlined that the above materials are not only subjected to significant loads (hip joint, knee joint), but also high friction forces due to their movement in contact with bones or cartilage [30,45].

Naveen et al. reported that hydrophilic materials were characterised by a higher protein adsorption capacity and therefore improved cell adhesion and proliferation [65]. However, our studies on the scaffold colonization over time and the ability of the composites show an inverse relationship. The cell viability is found to be highest for the 0.5Dic-oliP(3HO) blend (on day 7 of in vitro biocompatibility study 76.0 ± 6.4%), characterized by the strongest hydrophobic properties among the prepared ceramic-polymer composites, containing modified oligomers. This is possibly due to the toxicity of DIC, not due to the hydrophobicity. Additionally, we can conclude that obtaining a similar value of surface free energy and wettability for all prepared composites does not mean a similar level of eukaryotic cell viability. More important parameters affecting viability seems to be surface roughness or production of specific mediators by the cell, but predominantly contact with toxic chemicals, as it is with DIC. For example, it was shown, that in hOB cell line studies, 10^−5^ M DIC concentration does not inhibit cell proliferation, stops the cell cycle in the G0/G1 phase and does not induce nor inhibit important mediators such as cyclin-dependent kinase inhibitor 1B (p27^Kip1^), cyclin D2 and cell division protein kinase 2 (Cdk2). In the case of osteoblast growth inhibition, the expression of p27^kip1^ is increased, cyclin D2 expression decreased and cdk2 levels decreased [7].

The dynamic and complex environment that surrounds living cells enables the collection and efficient transport of signals necessary for their functioning. Complex interactions occurring at the cell–cell and cell-substrate levels affect the ability to adsorb proteins responsible for the regeneration of the appropriate tissue. However, an undoubted role in the abovementioned interactions is played by parameters relating to the surface characteristics—the as-mentioned wettability, surface energy, but also surface topography, its roughness as well as porosity and pore size distribution [66]. Taking into account only the topographic parameters of materials, i.e., roughness, it may have a different impact on the behaviour of living cells in the tested models [21]. For example, if the material is used as a bone regeneration scaffold, its surface structure should favour adhesion, proliferation and differentiation of the osteoblast, while the same parameters should be inhibitory for fibroblasts. This was confirmed in studies into changes of the topographic parameters of the TiO_2_ surface consisting of micropites and nanodules described by Kubo et al. Moreover, a better adhesion and proliferation of osteoblasts was observed in materials characterized by a higher surface roughness [67]. A similar relationship was observed in our research. Among the composites containing various doses of DIC-modified oligomers, the 0.5Dic-oliP(3HO) composite with the highest roughness (53.42 nm) showed the highest cell viability in the seven-day direct biocompatibility experiment (76.0 ± 6.4%).

As shown by Sadowska et al., the sintered disc β-TCP surface roughness was 1.33 ± 0.23 µm [68]. This result is several times higher than that obtained in our study. However, it should be remembered that infiltration of the polymer into the ceramic sponges results in the formation of a polymer layer on the surface of the prepared composite. According to our previous studies, the thickness of the polymer layer on the surface of the composite infiltrated with a 5% *w*/*v* P(3HO) solution was about 30 µm [17]. Therefore, the roughness of the composite will depend on the surface layer, which is a polymer or polymeric blend. Remembering that the unmodified P(3HO) thin films had a roughness lower (13.2 ± 2.8 nm) than the highest roughness value obtained in this study for the 0.5Dic-oliP (3HO) blend (53.42 nm), the TCP/P(3HO) composite should be characterized by weaker adhesion and colonization of the MC3T3-E1 cell line compared to the TCP/0.5Dic-oliP(3HO) composite. However, our research showed an inverse relationship. Cell viability on the 7^th^ day of the direct biocompatibility test was higher for TCP/P(3HO) (97.1 ± 4.4%) compared to TCP/0.5Dic-oliP(3HO) (76.0 ± 6.4%). In view of the above, the influence of characteristic markers and proteins involved in the inflammatory reaction of the cell lines studied should also be considered in future studies.

The analysis of literature data showed that DIC and other drugs from the NSAIDs group inhibits the viability of osteoblast cell lines—both MC3T3-E1 and MG63 as well as in the rabbit model—effectively preventing the processes associated with bone tissue regeneration [69,70,71]. In our study, the materials containing half of the therapeutic dose of chemically bonded DIC showed a cell viability over 70%, which, according to ISO 10993-5, confirms an absence of a cytotoxic effect. For the materials containing an amount of DIC equal or double the therapeutic dosage, a toxic effect could be detected. In other research, an inverse relationship in the case of cell scaffolds made of (P(3HB)-co-(3HV))/polyaniline composites with chemically bounded curcumin was shown [33]. Here, the in vitro biocompatibility analysis on the NIH 3T3 mouse fibroblast cell line resulted in better adhesion, adsorption and migration of cells on the surface of polymers with chemically bonded curcumin molecules. These results were associated with a change of roughness of the surface and the presence of permeating pores.

In the case of the in vitro biocompatibility studies, the results showed a decrease in cell viability, indicating a cytotoxic effect on the 3rd day of the experiment. However, it should be pointed out that the in vitro biocompatibility experiment was carried out under static conditions, and the culture medium was changed on the 3rd day. On the contrary, under natural conditions in the human body, a continuous circulation of body fluid occurs. The flow of blood and other fluids would undoubtedly reduce the concentration of the eluted drug, thus reducing the cytotoxic effect. Therefore, we may postulate that analysis in a specially designed bioreactor, with a continuous exchange of cultured medium, could provide a realistic picture of the cytotoxicity of the investigated materials [72]. The incorporation of DIC in the scaffolds was investigated to overcome the negative side effects associated with the systematic delivery of such a drug. Nevertheless, further studies should be conducted to investigate the therapeutic effectiveness of the amount of the drug released, through the quantification of the drug released and the analysis of the in vitro effect on the cells in terms of inflammation markers (e.g., prostaglandin E2 and nitric oxide production) [5].

## 5. Conclusions

Chemical modification of polymers and molecules derived from their decomposition is a promising approach, which offers new functionalities that could not be provided by unmodified molecules. In this work, composites based on tricalcium phosphates and polyhydroxyalkanoates with covalently attached molecules of non-steroidal anti-inflammatory drugs are obtained. We have confirmed polymer modification using several physicochemical methods (i.e., NMR, IR and XPS). Moreover, the analysis of wettability of films using prepared blends has shown an increasing hydrophilicity of materials as a function of the number of modified oligomers. AFM analysis of the prepared Fin-Dic-oliP(3HO)-based blends showed a different surface topography, which was dependent on the concentration of the modified oligomers used. Additionally, the surface roughness analysis showed that the 0.5Dic-oli(P3HO) blend is characterized by the highest roughness, which affects the adhesion and colonization of the MC3T3-E1 cell line on the prepared composites. Moreover, the results obtained from a physicochemical analysis, in combination with biological tests, allowed us to perform an in-depth analysis of scaffolds with potential use in bone tissue engineering. Furthermore, we have performed studies related to indirect cytotoxicity and the determination of in vitro biocompatibility behaviour, as well as the rate of cell colonization on the scaffolds over time, in a mouse pre-osteoblast cell line model (MC3T3-E1). The in vitro biocompatibility studies show no cytotoxicity effect for materials containing a half dose of the chemically bounded DIC (0.5Dic-oliP(3HO)). Thus, we can conclude that the oligomeric P(3HO) modified with DIC can provide a useful medical platform that opens up new therapeutic possibilities. Controlled release for long-term protection against chronic inflammation is essential and this is of particular importance in materials intended for the regeneration of bone defects, such as macroporous scaffolds, which are exposed to the occurrence of continual inflammation after implantation, associated with the human body immune system reaction. The use of covalently-linked NSAIDs such as DIC in this type of bone substitute is of crucial importance. It reduces the risk of rejection of the implanted foreign body. What is more, the use of biocompatible and bioactive materials such as TCP and P(3HO) provides a dual action: ceramic component due to its structural similarity to natural bone secures regeneration, while P(3HO) during natural enzymatic and physical hydrolysis processes, degrades to 3-(*R*)-hydroxycarboxylic acids, thus nourishing the implantation site.

## Figures and Tables

**Table 1:** The reaction mixture compounds per 1 g of blends.

Name	Fin-Dic-oliP(3HO)	P(3HO)
2Dic-oliP(3HO)	0.97	0.03
1Dic-oliP(3HO)	0.48	0.52
0.5Dic-oliP(3HO)	0.24	0.76

**Table 2:** Surface elemental compositions of P(3HO) and Fin-Dic-oliP(3HO).

	C (%)	O (%)	O/C Ratio	N (%)	Cl (%)
P(3HO)	77.25	22.75	0.29	n.d.	n.d.
Fin-Dic-oliP(3HO)	79.27	20.11	0.25	0.46	0.17

Where: n.d.—not detected.

**Table 3:** Physicochemical analysis of materials obtained and used in this study. The results are presented as mean ± SD (*n* = 3).

	M_n_ [kDa]	M_w_ [kDa]	Dispersity Index	T_onset_ (°C)	T_DTG_ (°C)	Residue (1%) (°C)	T_g_ (°C)	T_m_ (°C)
P(3HO) [21]	73	137	1.88	279	296	306	−41	53
Fin-Dic-oliP(3HO)	4.93 ± 0.0	8.21 ± 0.12	1.66 ± 0.02	285	293	298	−30	-
2Dic-oliP(3HO)	4.93 ± 0.0	8.21 ± 0.12	1.66 ± 0.02	285	293	367	−29	-
1Dic-oliP(3HO)	78.88 ± 4.55	133.74 ± 4.81	1.70 ± 0.04	280	294	305	−40	-
0.5Dic-oliP(3HO)	79.59 ± 0.12	139.26 ± 0.00	1.75 ± 0.00	281	288	308	−40	50
DIC	N/A	N/A	N/A	266	297	307	10	177

Where: N/A—not assayed.

## Supplementary Materials

Supplementary materials can be found at https://www.mdpi.com/1422-0067/21/24/9452/s1. Figure S1: NMR spectrum of the post-synthesis sample; Figure S2: The structures of compounds confirmed by NMR analysis; Figure S3: IR spectra of the investigated samples; Figure S4: The XPS survey spectra of the P(3HO) and Fin-Dic-oliP(3HO); Figure S5: The deconvoluted O 1s spectrum for P(3HO) (A) and for Fin-Dic-oliP(3HO) (B); Figure S6: TGA analysis of the investigated materials; Figure S7: Polar component (A) and dispersive component (B) of surface energy; Figure S8: The cell viability on the 3rd day of direct cytotoxicity test; Figure S9. SEM micrographs of TCP/1Dic-oliP(3HO) and TCP/2Dic-oliP(3HO) at day 1 and day 7 of incubations with cells. Table S1: Intensities of the fitted O 1s peaks; Table S2: Intensities of the fitted C 1s peaks.

## Abbreviations

0.5Dic-oliP(3HO)	Blend containing half doses of active substance per 1 g of polymer
1Dic-oliP(3HO)	Blend containing one dose of active substance per 1 g of polymer
1H NMR	Proton nuclear magnetic resonance
2Dic-oliP(3HO)	Blend containing two doses of active substance per 1 g of polymer
ACP	Amorphous tricalcium phosphate
ATR-FTIR	Attenuated total reflection Fourier transform infrared
C2C12	mouse myoblast cell line
Cdk2	Cell division protein kinase 2
DI	Dispersity index
DIC	Diclofenac acid
DSC	Differential scanning calorimetry
Fin-Dic-oliP(3HO))	Oligo-diclofenac conjugates
GPC	Gel permeation chromatography
HAp	Hydroxyapatite
hOBs	Human osteoblasts
MC3T3-E1	The mouse calvaria-derived pre-osteoblastic cell line
mcl-PHAs	Medium chain length polyhydroxyalkanoates
MEF3T3	Mouse embryonic fibroblast cell line
MG-63	osteosarcoma cell line
NIH 3T3	Mouse fibroblast cell line
NSAIDs	Non-steroidal anti-inflammatory drugs
P(3HB-co-4HB)	Copolymer of 3-hydroxybutyric acid and 4-hydroxybutyric acid
P(3HB)-co-(3HV)	Copolymer of 3-hydroxybutyric acid and 3-hydroxyvaleric acid
P(3HO)	Poly(3-hydroxyoctanoate)
p27Kip1	Cyclin-dependent kinase inhibitor 1B
PCL	Poly(caprolactone)
PEG	Poly(ethylene glycol)
PHAs	Polyhydroxyalkanoates
PLGA	Copolymer of dl-lactic acid and glycolic acid
PLLA	Poly(l-lactic acid)
PS	Polystyrene
PVAB	Copolymer of polyvinyl alcohol and boric acid
SAPAE	Copolymer of salicylic acid and succinic anhydride
SEM	Scanning electron microscopy
Sq	Root mean square roughness
TCP	Tricalcium phosphate
Tg	Glass transition temperature
TGA	Thermogravimetric analysis
Tm	Melting temperature
Tonset	Onset of thermal decomposition process
XPS	X-ray photoelectron spectroscopy
α-TCP	Alpha-tricalcium phosphate
β-TCP	Beta-tricalcium phosphate
