# Supplementary material for: Physicochemical and Biological Characterisation of Diclofenac Oligomeric Poly(3-hydroxyoctanoate) Hybrids as β-TCP Ceramics Modifiers for Bone Tissue Regeneration
Source: Int J Mol Sci. 2020 Dec 11;21(24):9452. doi: 10.3390/ijms21249452 (PMC7763618; doi:10.3390/ijms21249452)
Supplement: Supplementary file 1 [file ijms-21-09452-s001.pdf]

Supplementary Materials

Physicochemical and Biological Characterisation of Diclofenac Oligomeric Poly(3-hydroxyoctanoate) Hybrids as β-TCP Ceramics Modifiers for Bone Tissue Regeneration

Katarzyna Haraźna [1,*], Ewelina Cichoń [2], Szymon Skibiński [2], Tomasz Witko [1], Daria Solarz [3], Iwona Kwiecień [4], Elena Marcello [5], Małgorzata Zimowska [1], Robert Socha [1], Ewa Szefer [6], Aneta Zima [2], Ipsita Roy [7], Konstantinos N. Raftopoulos [6], Krzysztof Pielichowski [6], Małgorzata Witko [1] and Maciej Guzik [1,*]

[1] Jerzy Haber Institute of Catalysis and Surface Chemistry Polish Academy of Sciences, Niezapominajek 8, 30-239 Kraków, Poland; tomasz.witko@ikifp.edu.pl (T.W.); Malgorzata.zimowska@ikifp.edu.pl (M.Z.); Robert.socha@ikifp.edu.pl (R.S.); malgorzata.witko@ikifp.edu.pl (M.W.)

[2] Faculty of Materials Science and Ceramics, AGH University of Science and Technology, 30 Mickiewicza Ave., 30-059 Kraków, Poland; ecichon@agh.edu.pl (E.C.); skibinski@agh.edu.pl (S.S.); azima@agh.edu.pl (A.Z.)

[3] Faculty of Physics, Astronomy and Applied Computer Science, Jagiellonian University, Lojasiewicza 11, 30-348 Kraków, Poland; daria.solarz@doctoral.uj.edu.pl

[4] Department of Physical Chemistry and Technology of Polymers, Silesian University of Technology, M. Strzody 9, 44-100 Gliwice, Poland; Iwona.Kwiecien@polsl.pl

[5] School of Life Sciences, College of Liberal Arts and Sciences, University of Westminster, New Cavendish Street, London W1W 6UW, UK; w1614733@my.westminster.ac.uk

[6] Department of Chemistry and Technology of Polymers, Cracow University of Technology, Warszawska 24, 31-155 Kraków, Poland; ewa.szefer@doktorant.pk.edu.pl (E.S.); konstantinos.raftopoulos@pk.edu.pl (K.N.R.); kpielich@pk.edu.pl (K.P.)

[7] Department of Materials Science and Engineering, University of Sheffield, Broad Lane, Sheffield S3 7HQ, UK; i.roy@sheffield.ac.uk

* Correspondence: Katarzyna.harazna@ikifp.edu.pl (K.H.); Maciej.guzik@ikifp.edu.pl (M.G.); Tel.: +48-12-639-5156 (K.H.); +48-12-639-5153 (M.G.)

Received: 8 October 2020; Accepted: 9 December 2020; Published: 11 December 2020

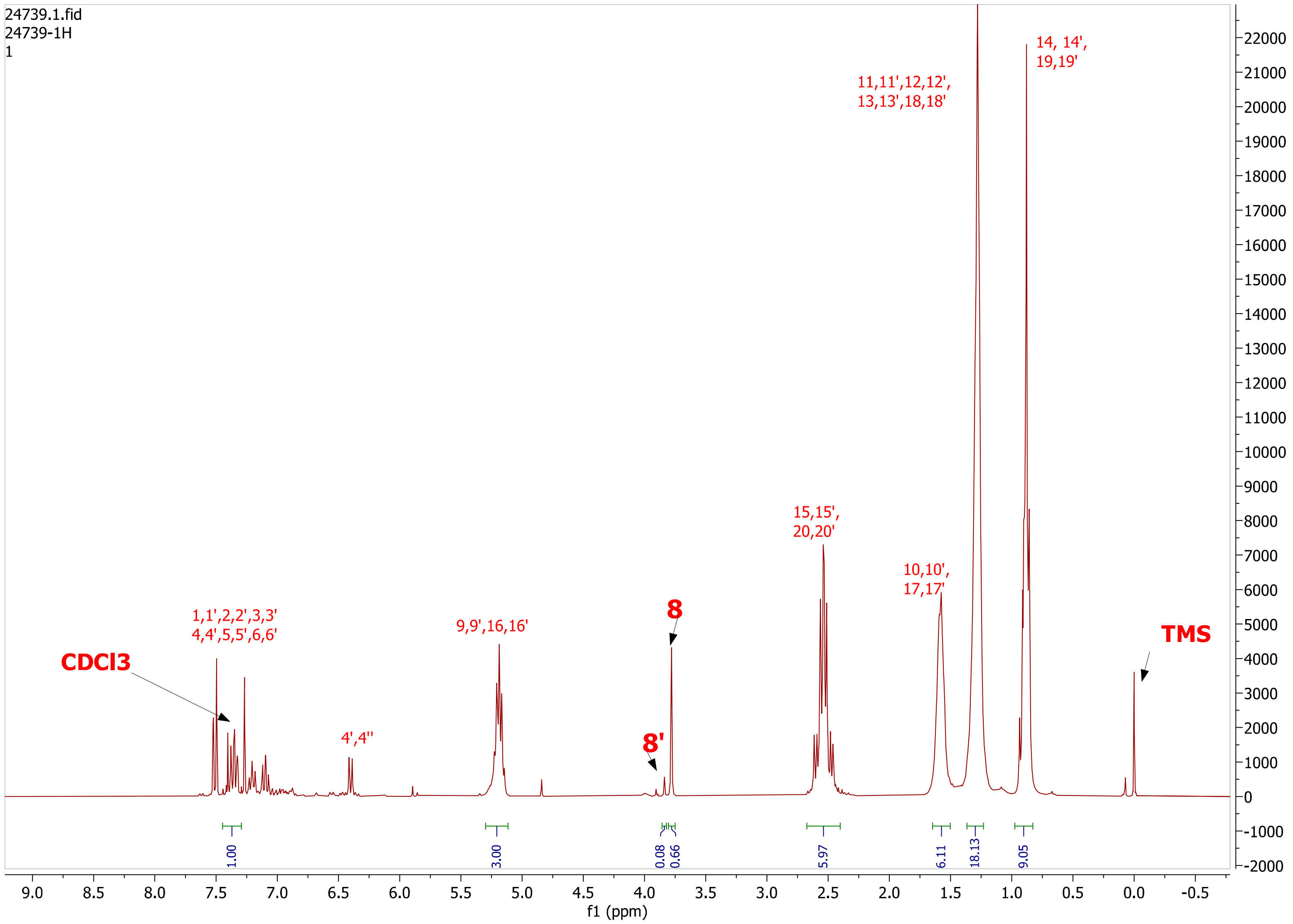

Figure S1. ^{1}H NMR spectrum of the post-synthesis sample. The corresponding atom numbers are located in Figure S2 at each of the species present in the mixture.

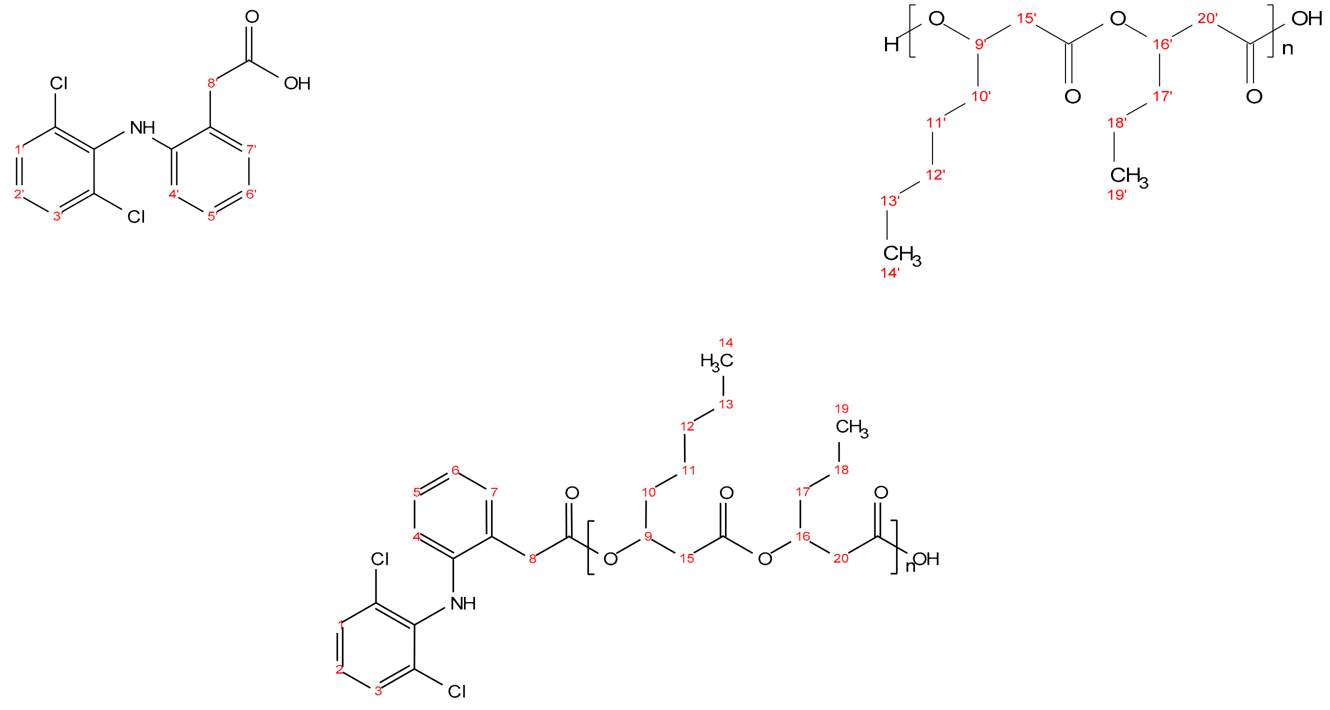

Figure S2. The structures of compounds confirmed by NMR analysis.

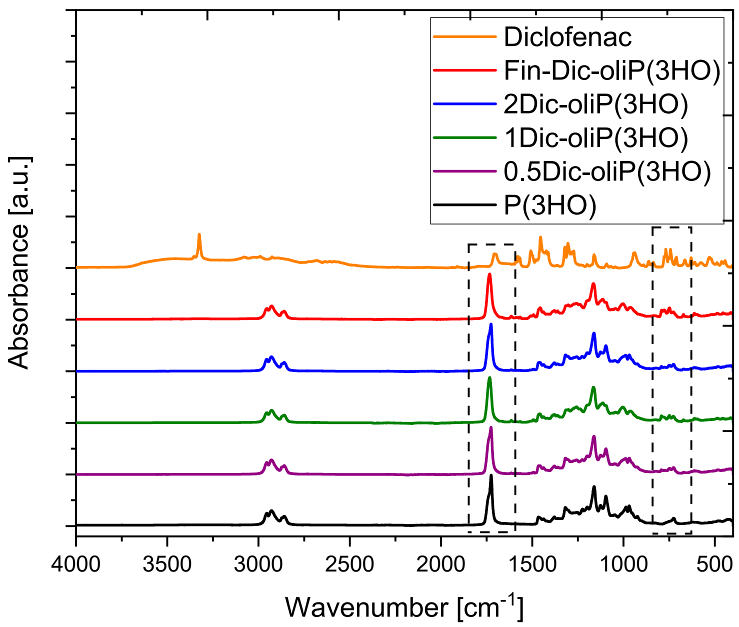

Figure S3. IR spectra of the investigated samples.

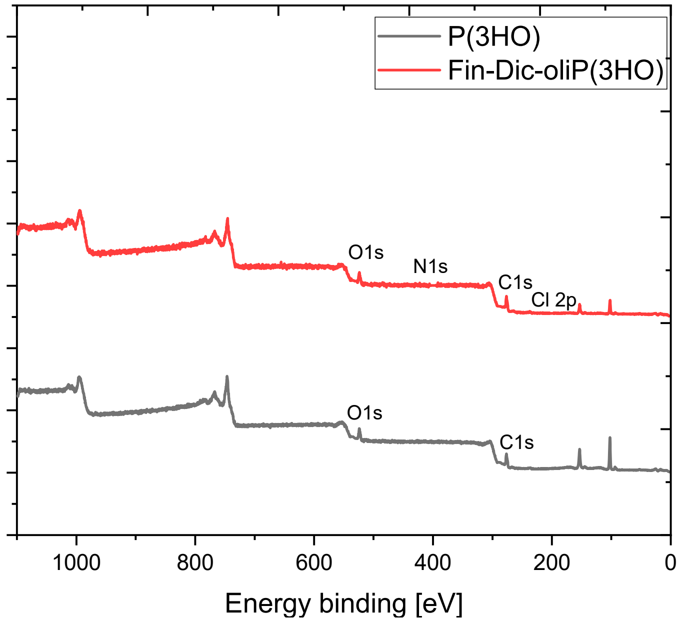

Figure S4. The XPS survey spectra of the P(3HO) and Fin-Dic-oliP(3HO).

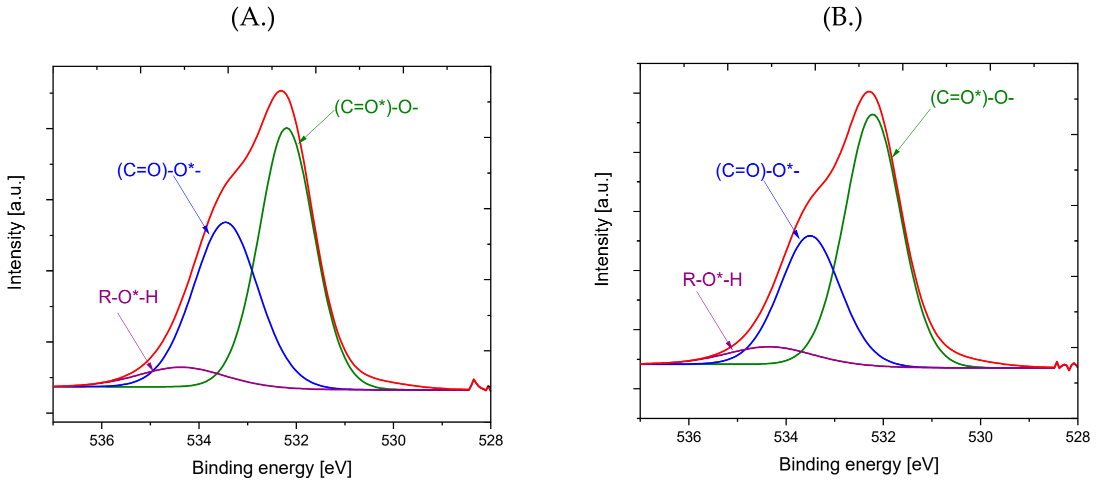

Figure S5. The deconvoluted O 1s spectrum for P(3HO) (**A**) and for Fin-Dic-oliP(3HO) (**B**).

Table S1. Intensities of the fitted O 1s peaks.

	Possition BE (eV)	Area (%)	Element state
	532.2	53.6	(C=O)-O*
P(3HO)	533.4	40.1	-(C=O*)-O-
	534.4	6.3	R-O*H
	532.2	60.4	(C=O*)-O-
Fin-Dic-oliP(3HO)	533.5	33.2	(C=O)-O*-
	534.3	6.4	R-O*H

Table S2. Intensities of the fitted C 1s peaks.

	Possition BE (eV)	Area (%)	Element state
P(3HO)	285.0	74.2	C-C
	286.4	12.7	C-O
	287.2	4.9	C=O
	289.2	8.2	COOH
Fin-Dic-oliP(3HO)	285.0	73.6	C-C
	286.3	10.7	C-O
	287.2	6.4	C=O + =C-N
	289.3	9.3	COOH

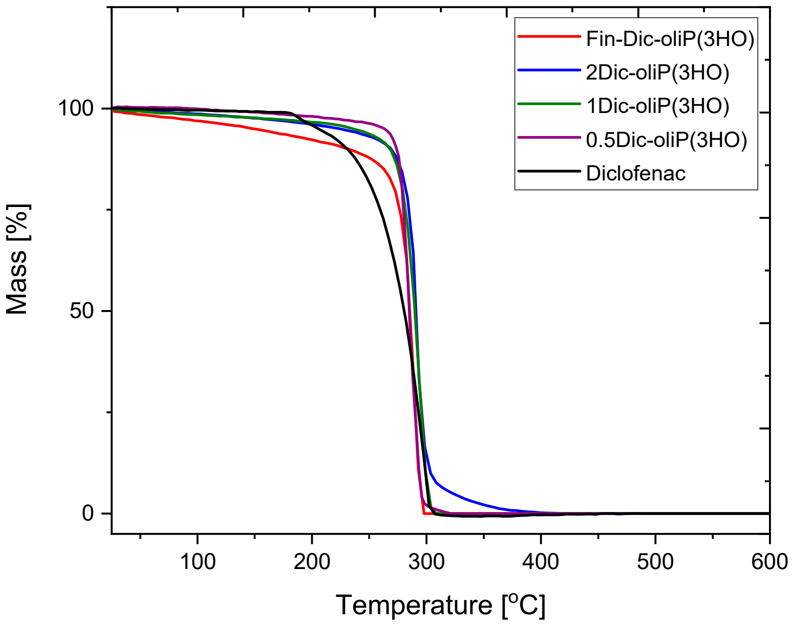

Figure S6. TGA analysis of the investigated materials.

(A.) (B.)

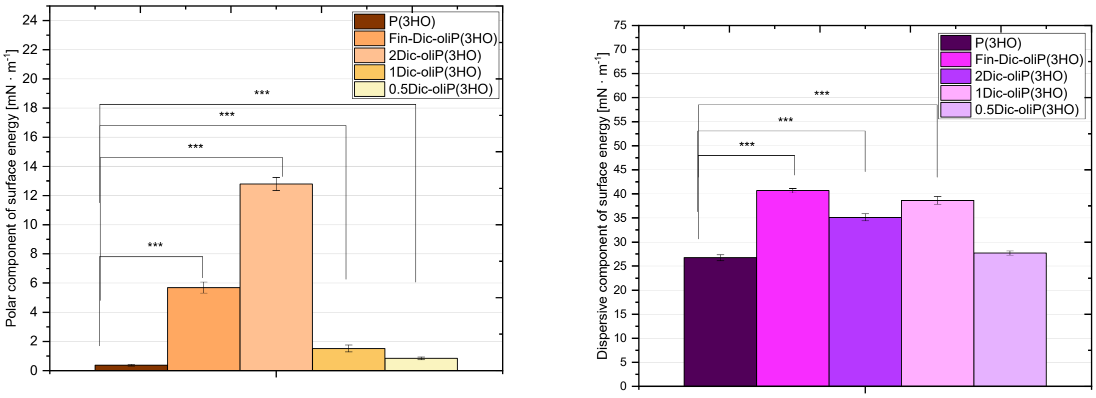

Figure S7. Polar component (**A**) and dispersive component (**B**) of surface energy (n = 36; error bars = ±SD). The results are statistically significant, where: * $p < 0.05$, ** p <0.01, *** p<0.001.

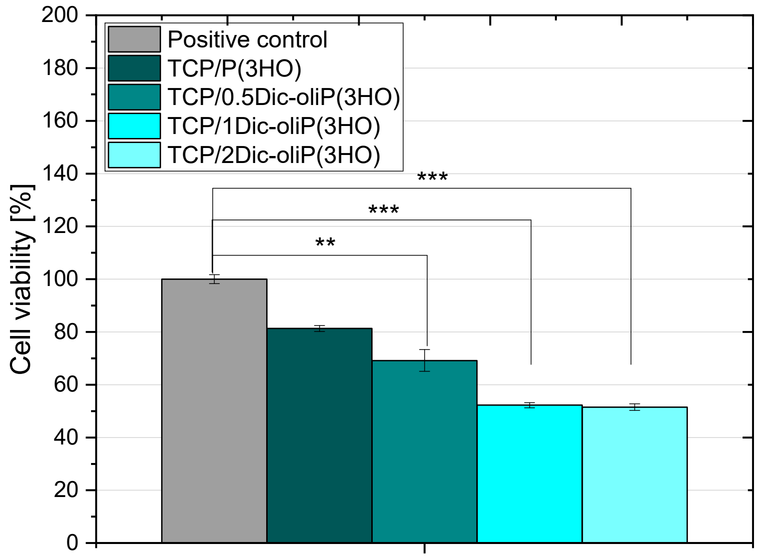

Figure S8. The cell viability on the 3rd day of direct cytotoxicity test ($n = 3$; error bars = ±SD). The results are statistically significant, where: ** $p < 0.01$, *** $p < 0.001$.

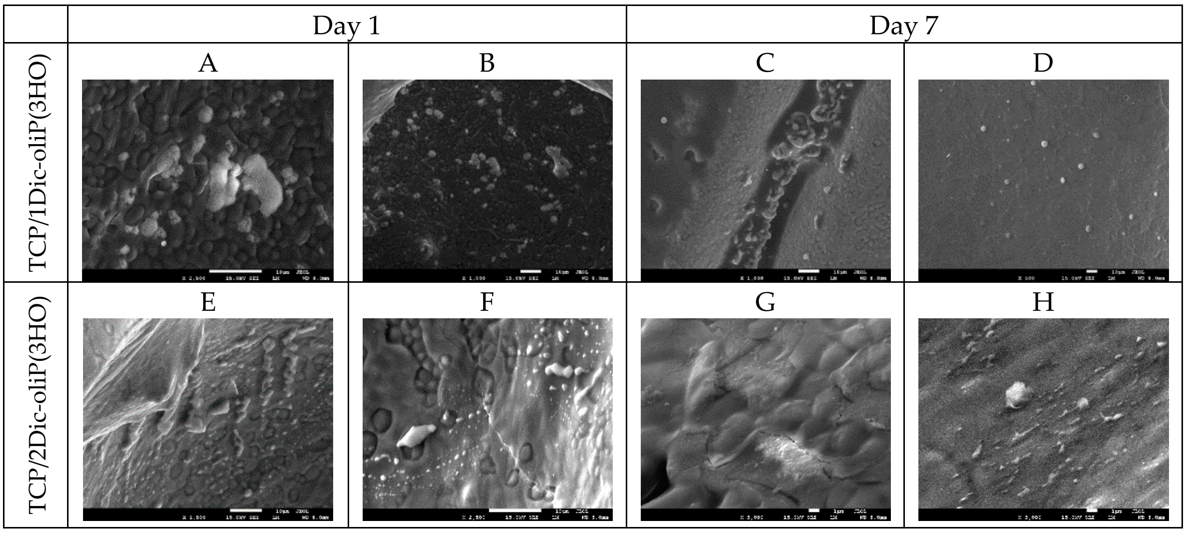

Figure S9. SEM micrographs of TCP/1Dic-oliP(3HO) and TCP/2Dic-oliP(3HO) at day 1 and day 7 of incubations with cells. Bars in A to F correspond to 10 μm, whereas in G and H to 1 μm.
